# A survey of anaesthetic management of regional anaesthesia for patients undergoing arthroscopic shoulder surgery in Australia

**DOI:** 10.1186/s12871-025-03459-3

**Published:** 2025-11-17

**Authors:** Clayton Y. X. Lam, Sam Whitehouse, Brigid Brown, Paul Maclure, Elise Kingston, Maxim Nagorny, Steve Wilson, Ruurd L. Jaarsma, D-Yin Lin

**Affiliations:** 1 Flinders Medical Centre, Department of Anaesthesia and Pain Medicine, Adelaide, SA Australia; 2 College of Medicine and Public Health, Flinders University, Adelaide, SA Australia; 3 Flinders Medical Centre, Department of Orthopaedic and Trauma Surgery, Adelaide, SA Australia; 4 Department of Anaesthesiology, Flinders Medical Centre, Flinders Drive, Bedford Park, SA 5042 Australia

**Keywords:** Regional Anaesthesia, Shoulder Arthroscopy, Ultrasonography, Interscalene, Superior Trunk, Suprascapular

## Abstract

**Background:**

The Australian and New Zealand College of Anaesthetists recommends standards of monitoring and conduct for regional anaesthesia but does not endorse specific regional techniques. While the interscalene block is the de-facto standard technique, there are numerous established and novel alternatives. Regional block choice and block-related variables (local anaesthetic type, dose, volume, adjuncts) are subject to individual and institutional practices.

**Methods:**

Using a 30-question survey, we aimed to create a snapshot of current regional anaesthesia practice for patients undergoing arthroscopic shoulder surgery across Australia and New Zealand.

**Results:**

Among 134 anaesthetist respondents, 85.1% routinely placed a regional block for arthroscopic shoulder surgery. 91.2% performed interscalene blocks. Ropivacaine 0.375% and 0.75% were the most common concentrations and type. Regional anaesthesia under light sedation and general anaesthesia were performed by 47.4% and 30.7% block-respondents respectively. 96.5% of block-respondents used ultrasonography. 49.1% of block-respondents recorded written consent. 86.0% always performed a consent/site/side check. Standard monitoring was variably applied for blood pressure, electrocardiography and end-tidal carbon dioxide monitoring. Postoperatively, patient-controlled analgesia was not routinely prescribed.

**Conclusion:**

Variation exists for regional anaesthesia techniques for arthroscopic shoulder surgery with most performing interscalene blocks with ultrasonography. This study highlights topics of debate including level of consciousness during block performance and overall adherence to monitoring recommendations, so as to ensure patient and anaesthetist safety is upheld.

**Clinical trial number:**

Not applicable.

**Supplementary Information:**

The online version contains supplementary material available at 10.1186/s12871-025-03459-3.

## Background

Arthroscopic shoulder surgeries are common elective procedures and often planned for same-day discharge. These surgeries can cause significant postoperative pain with inadequate analgesia adversely impacting the patient’s rehabilitation and recovery [1]. This can then lead to unintended hospital re-admissions or delayed discharges for same-day surgeries [2]. Upper limb regional nerve blocks have demonstrated benefit as part of a multimodal analgesia regime in reducing peri-operative analgesia requirement and post-operative pain from shoulder surgery [3, 4]. 

The Australian and New Zealand College of Anaesthetists (ANZCA) maintains clear standards of care relating to the provision, monitoring and management of regional anaesthesia [5]. Many other major worldwide anaesthesia and regional anaesthesia societies publish their own educational resources, including the New York School of Regional Anesthesia (NYSORA) and the British Journal of Anaesthesia [6, 7]. 

Thus far, there have been no recent point-prevalence study conducted to establish regional anaesthesia choice by any general cohort of anaesthetic specialists. Numerous regional nerve blocks have been suggested as an alternative to the commonly performed interscalene block, including the superior trunk block, suprascapular block, axillary nerve block, or a combination of multiple regional techniques [8–10]. In addition to block choice, a wide variety of block-related variables exist (local anaesthetic type, volumes, adjuvant medications and site identification techniques) within individual clinical practice. However, the relative frequencies of use for each type of block and the various block related variables are unknown.

The authors aimed to determine how a representative cohort of anaesthetists recruited over eight months in 2024 across Australia and New Zealand, routinely implemented regional anaesthesia techniques for patients presenting for arthroscopic shoulder surgery.

## Methods

Multi-centre ethics approval was obtained from the South Australia Local Health Network Office for Research Ethics Committee (SALHN HREC OFR 78.20).

The survey was originally formulated as a consensus amongst authors C.Y.X.L, S.W, B.B, P.M, E.K, M.N, S.W, and R.L.J. To minimise groupthink and ensure blinding, the aforementioned authors were asked individually to provide their opinions regarding pre-, peri- and post-procedural considerations relating to regional anaesthesia for arthroscopic shoulder surgery. Feedback analysis was performed regarding any disagreements or confusion in question wording, which was then escalated to all authors for review and improvement. Authors C.Y.X.L and S.W then reviewed concerns raised from question wording. Multiple rounds of formulation and revision were carried out under the coordination of author S.W and a revised 30-question survey was agreed upon by the same original listed author panel for distribution.

Results were collected by an anonymised online survey questionnaire utilising Microsoft Forms (Microsoft, Redmond, Washington, USA). Eligibility criteria were established prior to commencement. Only specialist anaesthetists and anaesthesia fellows (ANZCA Professional Fellowship Year candidates) who were the primary anaesthetist for more than one patient over the past year undergoing arthroscopic shoulder surgery were eligible to participate. All survey results were anonymous and analysed as pooled results. While all questions were compulsory, comments in designated text fields were optional.

The survey introduction articulated the aim, inclusion criteria and a statement that voluntary anonymous participation would be considered as implied consent. There were 30 questions in this structed questionnaire, with 26 multiple-choice questions and four free-text boxes. A copy of the survey has been included in Appendix 1.

The survey questions addressed several key themes, involving:

1. Three items related to respondent demographics (including overall experience and primary type of practice).
2. Seven items related to pre-procedural considerations (routine placement of regional block for arthroscopic shoulder surgery, informed consent, block site marking/side check, sedation level, monitoring, intralipid accessibility).
3. 15 related to peri-procedural considerations (Block of choice, choice of primary local anaesthetic agent +/- block combinations, use of adjuvants, choice of equipment/aids to improve block performance).
4. Five related to post-procedural considerations (Use of patient-controlled analgesia (PCA), discharge opiate scripts, post-operative follow-up, post-operative complications).

From January to August 2024, study investigators distributed the survey to current registered Australian Society of Anaesthetists (ASA) Professional Group Members for them to further share with their fellow anaesthetic colleagues at their respective institutions throughout Australia and New Zealand. The survey was shared via a Quick Response (QR) code and weblink for which respondents were directed to the survey for completion. Authors C.Y.X.L and S.W collected and reviewed the pooled data from respondents.

Analysis of the pooled survey results were performed using Stata Version 18.0 (StataCorp, Texas, USA). Means ± standard deviation was calculated from continuous variables, and categorical variables were expressed as counts and percentages.

## Results

There were 134 responses with most respondents from Australian metropolitan hospitals (91.8%, 123/134) and reporting over five years of clinical anaesthesia experience (79.9%, 107/134). There were no respondents from practitioners working in New Zealand hospitals. Table 1 categorises the respondents’ demographics in further detail.

**Table 1:** Distribution of respondent demographics for arthroscopic shoulder surgery survey

	All respondents (*n* = 134)	
	*n*	%
Current employment level		
Consultant	133	99.3
Fellow	1	0.7
Experience (years)		
0–5	27	20.2
6–10	30	22.4
11–15	16	12.0
16–20	22	16.4
21–25	21	15.7
> 25	18	13.4
Primary practice		
Private	56	41.8
Public	78	58.2
Current location (State) of work		
New South Wales	29	21.6
Queensland	11	8.2
SA	61	45.5
Tasmania	17	12.7
Victoria	16	11.9
Rurality of hospital work		
Metropolitan	123	91.8
Regional	11	8.2
Routinely place a regional nerve block for arthroscopic shoulder surgery		
Yes	114	85.1
No	20	14.9

Area of primary practice was mainly in Australian public hospitals (58.2%, 78/134), with a wider demographic of respondents working in South Australia (SA) (45.5%, 61/134) relative to other states.

Of the 134 respondents, 85.1% (114/134) reported they would routinely place a regional block for arthroscopic shoulder surgery where no contraindications to regional anaesthesia existed.

For the 14.9% (20/134) respondents who reported they would not routinely place a regional block, the following main themes were observed:

1. Decision would be made on a case-by-case basis following discussion with surgeon and/or patient (7/20, 35.0%).
2. Did not feel the surgery causes enough pain to warrant a regional block (6/20, 30.0%).
3. Not confident in regional anaesthesia techniques (4/20, 20.0%).
4. Others* (3/20, 15.0%).

*Did not fall into any of the above categories (for example: afraid of complications associated with regional anaesthesia, patient disposition).

### Distribution of respondent demographics for arthroscopic shoulder surgery survey

A summary of pre-procedural factors prior to regional anaesthesia are outlined in Table 2. Individuals who performed regional blocks were considered “block-respondents.” Informed written consent was not always obtained amongst block-respondents, with 50.9% (58/114) reporting this as routine. Prior to performing the regional nerve block, 80.7% (92/114) of block-respondents reported they always ensured the block site/side was marked, and 86.0% (98/114) of block-respondents always performed a consent/site/side check. Level of patient sedation whilst performing the regional block varied amongst operators, with 57.9% (66/114) of block-respondents using light to no sedation peri-procedurally. Conversely, 30.7% (35/114) of block-respondents reported routinely placing their regional block under general anaesthesia.

**Table 2:** Pre-procedural considerations prior to attempting regional anaesthesia for arthroscopic shoulder surgery

	For respondents who would routinely place a regional nerve block for arthroscopic shoulder surgery (*n* = 114)							
	*n*				%			
Informed written consent								
Yes	56				49.1			
No	58				50.9			
Level of Sedation								
None	12				10.5			
Light	54				47.4			
Moderate	12				10.5			
Heavy	1				0.9			
General Anaesthesia	35				30.7			
Preparation prior to performing nerve block								
	Never		Sometimes		Often		Always	
	n	%	n	%	n	%	n	%
Ensure block site/side is marked	10	8.8	6	5.2	6	5.3	92	80.7
Personally mark the block site	61	53.5	28	24.6	5	4.4	20	17.5
Perform a consent/site/side check	2	1.8	2	1.8	12	10.5	98	86.0
Monitoring								
	Never		Sometimes		Often		Always	
	n	%	n	%	n	%	n	%
Dedicated Trained Assistant	2	1.8	11	9.7	15	13.2	86	75.4
Place an IV cannula prior to commencement of block	0	0	0	0	1	0.9	113	99.1
Monitor oxygen saturations	3	2.6	19	16.7	12	10.5	80	70.2
Monitor blood pressure	17	14.9	23	20.2	16	14.0	58	50.9
Monitor electrocardiography	31	27.2	30	26.3	15	13.2	38	33.3
Monitor respiratory rate or end-tidal carbon dioxide	50	43.9	24	21.1	9	7.9	31	27.2
Accessibility to Intralipid 20%								
	n				%			
Yes	108				94.7			
No	6				5.3			

Patient monitoring during the procedure differed amongst block-respondents, with 70.2% (80/114) always monitoring oxygen saturations with pulse oximetry, 50.9% (58/114) always monitoring blood pressure, 33.3% (38/114) always monitoring electrocardiography, and 27.2% (31/114) always monitoring respiratory rate/end-tidal carbon dioxide. Almost all block-respondents (113/114, 99.1%) ensured an intravenous cannula is placed prior to commencement of attempting the regional block. Six block-respondents (5.26%, 108/114) reported they would be unable to quickly access an appropriate dose of Intralipid 20% in the event of an emergency.

### Pre-procedural considerations prior to attempting regional anaesthesia for arthroscopic shoulder surgery

Table 3 highlights the peri-procedural considerations and details related to choice of regional anaesthesia block for arthroscopic shoulder surgery. Most block-respondents (91.2%, 104/114) routinely used the interscalene block for patients undergoing arthroscopic shoulder surgery. This was followed by the superior trunk block (8.8%, 10/114). Most block-respondents used ropivacaine as their local anaesthesia (LA) of choice at concentrations of 0.375% (40.4%. 46/114) and 0.75% (37.7%, 43/114). Mean LA volume administered was 15 ml, with a range of volumes noted from 5 ml to 30 ml. Seven block-respondents (6.1%) used a mix of local anaesthetics, specifically 1% lignocaine with or without adrenaline, in combination with ropivacaine. 43 block-respondents (37.7%) routinely added adjuvants into their block injectate, with dexamethasone being the most common (31/43, 72.1%). Other reported adjuvants included clonidine, dexmedetomidine and buprenorphine.

**Table 3:** Peri-procedural considerations and details relating to choice of regional anaesthesia block for arthroscopic shoulder surgery

		For respondents who would routinely place a regional nerve block for arthroscopic shoulder surgery (*n* = 114)							
		*n*				%			
Choice of regional nerve block (multiple responses accepted)									
Interscalene block		104				91.2			
Superior trunk block		10				8.8			
Axillary nerve block		3				2.6			
Intercostobrachial nerve block		2				1.8			
Suprascapular nerve block		5				4.4			
Supraclavicular nerve block		5				4.4			
Other		1				0.9			
Primary local anaesthetic agent for choice of regional block									
Ropivacaine	0.2%	3				2.6			
	0.25%	1				0.9			
	0.375%	46				40.4			
	0.5%	8				7.0			
	0.75%	43				37.7			
	1.0%	7				6.1			
	2.0%	1 (likely error)				0.9 (likely error)			
Bupivacaine	0.2%	1 (likely error)				0.9 (likely error)			
	0.375%	2 (likely error)				1.8 (likely error)			
Bupivacaine with adrenaline	0.5%	2				1.8			
Volume (mL) of local anaesthetic administered									
5–9		9				7.9			
10–14		47				41.2			
15–19		28				24.6			
20–24		28				24.6			
> 25		2				1.8			
Mixture of primary local anaesthetic agent with second local anaesthetic									
Yes		7				6.1			
No		107				93.9			
Routinely add adjuvants in block injectate									
Yes		43				37.7			
No		71				62.3			
Routinely use aids to assist in block performance									
		Never		Sometimes		Often		Always	
		n	%	n	%	n	%	n	%
Ultrasound		1	0.9	2	1.8	1	0.9	110	96.5
Nerve stimulator		90	79.0	16	14.0	7	6.1	1	0.9
Injector pressure monitor		108	94.7	5	4.4	1	0.9	0	0
Leave nerve catheter in-situ									
		n				%			
Never		78				68.4			
Sometimes		33				29.0			
Often		2				1.8			
Always		1				0.9			

The majority of block-respondents (96.5%, 110/114) utilised ultrasonography to assist in block performance with the syringe injection often performed by an assistant (68.4%, 78/114). A 50 mm echogenic needle was most commonly chosen (79.0%, 90/114) with only a small proportion of block-respondents at 0.97% (3/114) often leaving a nerve catheter in-situ.

### Peri-procedural considerations and details relating to choice of regional anaesthesia block for arthroscopic shoulder surgery

Most respondents reported routine anaesthesia management was not influenced if the case was a planned day surgery or overnight admission (89.6%, 120/134). For respondents who would alter management (10.5%, 14/134), the following themes were reported:

1. Consider no regional block (28.6%, 4/14).
2. Consider no nerve catheter (21.4%, 3/14).
3. Consider a lower concentration of local anaesthetic (14.3%, 2/14).
4. Others* (35.7%, 5/14).

*Did not fall into any of the above categories.

Routine charting of a patient-controlled analgesia (PCA) was infrequent (13.4%, 18/134) for post-operative pain management. Most respondents (67.9%, 91/134) reported prescribing a discharge opiate script. Most respondents (62.7%, 84/134) did not routinely follow up these patients (in person or over the phone). Six respondents (4.5%, 6/134) reported detecting and managing post-procedure complications: four of these were neuropraxia (66.7%, 4/6), one was intractable pain (16.7%, 1/6), and one was non-anaesthetic related (16.7%, 1/6). Post-procedural considerations and follow up are further detailed in Table 4.

**Table 4:** Post-procedural considerations and post-operative anaesthesia-related complications from arthroscopic shoulder surgery

	All respondents (*n* = 134)	
	*n*	%
Would keep anaesthetic management the same based regardless of being day surgery procedure or overnight admission		
Yes	120	89.6
No	14	10.4
Routinely prescribe an intravenous patient-controlled analgesia pump		
Yes	18	13.4
No	119	88.9
Routinely provide discharge opiate script		
Yes	91	67.9
No	43	32.1
Routinely follow up post-operative arthroscopic shoulder surgery patients (in person/over phone)		
Yes	50	37.3
No	84	62.7
Found a significant post-operative complication through follow-up		
Yes	6	4.5
No	128	95.5

### Post-procedural considerations and post-operative anaesthesia-related complications from arthroscopic shoulder surgery

## Discussion

Of 134 respondents, 114 would routinely place a regional block to enhance analgesia for arthroscopic shoulder surgery. While the responses show a clear consensus around block choice and key block-related variables, they also demonstrate some variation in practice.

### Pre-procedural considerations

#### Informed written consent

Obtaining written informed consent prior to performing the regional techniques was inconsistent. Although the survey failed to capture rate of response for non-written (verbal) informed consent, the authors hope that the aggregate of both measures would approach 100%. ANZCA’s position statement (PS26) relating to informed consent for anaesthesia and sedation 2021, [11] acknowledges the importance of documenting significant consent details within the patient’s notes and discussion of relevant material risks [11]. However, local, institutional and state practice guidelines differ in the legal and expected standards of formal documentation of consent. Given the risks associated with regional blocks, maintaining detailed notes ensures best practice and transparency between the patient and provider [11]. 

#### Level of sedation

The degree of planned sedation for patients during the regional nerve block varied amongst respondents. The respondents tell the tale of two extremes, with blocks performed most under either light or no sedation (57.9%, 66/114), or with the patient under general anaesthesia (30.7%, 35/114). Conscious sedation for adults undergoing regional anaesthesia is postulated to improve safety and success of peripheral nerve block placement,[12] as an awake or semi-awake patient can act as a real-time monitor for paraesthesia associated with needle-to-nerve proximity. This allows the proceduralist to reposition the needle before or prior to intraneural injection [13]. However, patient discomfort and movement can complicate needle placement leading to inadvertent direct needle trauma and reducing patient satisfaction. ANZCA guidelines do not make recommendations regarding level of sedation for regional block performance [14]. The Second ASRA Practice Advisory on Neurologic Complications Associated With Regional Anaesthesia and Pain Medicine (2015) makes a specific recommendation that neuraxial anaesthesia be rarely performed in patients under general anaesthesia or deep sedation [15]. However, this is a class III recommendation only (*“the usefulnesss of the recommendation is limited by absent or conflicting evidence and/or divergent expert opinion”*) and may not be applicable to other major regional techniques [15]. 

#### Preparation and patient monitoring

Although most block-respondents routinely performed a thorough review of patient consent, correct site and side prior to administering regional anaesthesia, around 15% do not. Wrong-site block incidence may be as frequent as 7.5 per 10 000 procedures, yet these iatrogenic injuries should be considered ‘never’ events and prevention should be a primary aim of all proceduralists [16]. Literature has highlighted the benefit of implementing a pre-procedural regional anaesthesia-specific checklist prior to performing major regional anaesthesia, specifically in potentially reducing the incidence of wrong-site nerve blocks [17]. Institutional uptake and inclusion into training programmes is likely to further increase the uptake of this simple evidence-based reform.

24.7% (28/114) of block-respondents do not always have a dedicated trained assistant when performing regional blocks for arthroscopic shoulder surgery. While staffing availability is not always within the proceduralist’s control, ANZCA guidelines state that initiation of major regional analgesia requires appropriate assistance to ensuring safe patient management and assist the anaesthetist in the event of an emergency [14, 18]. 

The extent of monitoring of patients undergoing regional anaesthesia for shoulder arthroscopy varied widely. While most monitor oxygen saturations and therefore heart rate via pulse oximetry, less than 50% routinely monitored blood pressure, electrocardiography, or respiratory rate/end-tidal carbon dioxide. ANZCA recommends regular blood pressure measurements alongside respiratory rate, and conscious state evaluation during major regional anaesthesia [14]. Given these parameters are part of standard monitoring for general anaesthesia, it is vital these are adhered to whenever regional techniques are being formed as routine deviation from ANZCA professional guidelines poses a potential increased risk to both patients and anaesthetists. However, a subsequent survey looking at the suggested monitoring alongside experienced safety complications by respondents during regional techniques, may assist with investigating reasons for the discrepancy between the self-reported practices of respondents in our survey and suggested ANZCA monitoring.

Only one block-respondent (0.9%) reported routinely performing nerve blocks without always having an intravenous cannula prior. Given the small but significant risk of complications requiring specific or supportive intravenous therapy, and given the reality that intravenous access is required for surgery, this response is hard to justify [14]. 

#### Accessibility to 20% intra-lipid

Local anaesthetic systemic toxicity (LAST) manifests as significant neurological and cardiovascular events with estimated severe toxicity is as high as 7.5 to 20 per 10 000 peripheral nerve blocks [19]. Early administration of 20% intralipid can be a life saving measure.[19] 5.3% (6/114) of block-respondents reported they would be unable to quickly access an appropriate intralipid dose in an emergency. There may be many reasons for this: some anaesthetists work across multiple sites and may not have considered availability of intralipid at one or more location; or it could represent a lack of contingency planning. Furthermore, some hospitals may not prioritised easy access to this antidote. Given the utility of intralipid for consequences related to LAST, [20] these should be made readily available alongside advance cardiac life support equipment where regional anaesthesia is practiced [14]. 

### Peri-procedural considerations

#### Choice of regional nerve block

91.2% of block-respondents (104/114) routinely performed an interscalene block for patients undergoing arthroscopic shoulder surgery. The interscalene block is generally accepted as the ‘gold-standard’ nerve block for shoulder surgery, providing complete anaesthetic block for most procedures [21]. The second most frequently reported block was the superior trunk block, a recently reported modification of the interscalene block with a more favourable adverse effect profile [22]. As the target for superior trunk block is more distal and lateral to the interscalene groove, there is a significant reduction in post-procedure phrenic nerve palsy rates and associated diaphragmatic paresis, whilst providing non-inferior surgical anaesthesia [22, 23]. This makes it ideal for patients in whom such an outcome would complicate respiratory function.

#### Primary local anaesthetic agent

Ropivacaine, at concentrations of 0.375% and 0.75%, is the preferred choice of primary local anaesthetic agent for regional anaesthesia. This is presumably due to its preferable cardiovascular risk profile in toxicity compared to other agents, like bupivacaine. Although no drug is specifically recommended by ANZCA guidelines, literature favours 0.75% ropivacaine for upper limb surgeries due to its rapid onset of sensory and motor blockade [24]. Preferred volumes of local anaesthetic administered were widely varied, with the majority of respondents reporting a dose of 10–14 ml. A recent study comparing 10 ml of 0.75% ropivacaine and 20 ml of 0.375% ropivacaine during an interscalene block showed that whilst the higher concentration potentially reduced post-operative opioid requirements in the first 24 h, it may not prolong the analgesic duration of the block.[25] 37.7% (43/114) of respondents would add an adjuvant to their block injectate, with 72.1% (31/43) of those who used adjuvants routinely adding dexamethasone with their local anaesthetic. This is consistent with current evidence showing dexamethasone can increase the quality and regional anaesthesia duration, especially for interscalene block [26]. Other adjuvants reported by respondents included the α−2 agonists clonidine and dexmedetomidine, which can both prolong duration of block at varying efficacies [27, 28]. 

#### Aids and equipment used to improve block performance

Easy access to equipment, including ultrasound and/or peripheral nerve stimulators are desirable adjuncts to improving success during regional anaesthesia [14]. Ultrasonography improves visualisation of the target nerve and needle-tip position, enabling improved local anaesthesia spread compared to landmark-based techniques [4]. Due to the general focus on ultrasound teaching within anaesthesia training programmes especially for regional anaesthesia, the use of nerve stimulators in clinical practice is minimal which is supported by the survey responses. The use of ultrasonography has also showed improved success rates amongst trainees, with improved block and imaging time [29]. 

Most respondents use a 50 mm echogenic needle for the procedure, with most relying on their assistant to perform syringe injection, although a significant plurality perform their own injections. Performing a further sub-descriptive analysis or secondary survey in future, specifically focusing on years of practice/experience, could better evaluate the distribution of uptake of ultrasonography use currently with regional techniques.

Few block-respondents (2.6%, 3/114) would place a nerve-catheter for this procedure type. This may be due to the numerous logistical challenges associated with the subsequent management, removal and follow up associated with this technique, especially in private practice. Furthermore, the superficial nature of the brachial plexus at interscalene level and the tendency for patients to rotate their neck in daily activities, [30] increases the likelihood of catheter dislodgement. Finally, the unnecessity of prolonged regional analgesia and the potential for prolonged hospital stay may reduce the provision of catheter-based techniques.

### Post-procedural considerations

Few respondents (13.4%, 18/134) chart an intravenous opiate PCA for post-operative pain management. However, most prescribe a discharge opiate for ongoing analgesia following discharge (67.9%, 91/134). This pattern may be partially attributable to arthroscopic shoulder procedures being generally day-stay procedures. Further research determining clinician preference relating to dose and amount of discharge opiate could help standardise management and reduce post-operative pain crises in the community. Conversely, it may be that opioid medications are supplied in excess, and this could judiciously be reduced in the context of the current opioid epidemic [31]. It should be mentioned that in many centres, it is the treating surgical team’s responsibility to prescribe discharge medications and this fact may have skewed the responses to this question.

## Limitations

Although this is a robust survey conducted across multiple sites nationally, limitations to this study must be acknowledged. Compared to the overall current number of anaesthetists across Australia, our sample size was relatively small with a larger prevalence of respondents from South Australia from where the study was run. Hence, this may influence generalisability of the data results. The nature of the study being a survey may introduce responder bias to answers collated and therefore affect the reliability of the conclusions able to be drawn. The small sample size from our survey may also make extrapolation of findings to wider direct clinical practice limited. The results also may be influenced by local standards based on the primary hospital of practice by the respondents. As the survey results were anonymous, including hospital of employment, clarification of certain responses was not possible.

## Conclusion

This multi-centre, cross-sectional study of anaesthetists across Australia has highlighted a variety of practices relating to regional anaesthesia for arthroscopic shoulder surgery. There was inconsistent documentation of written informed consent of the block being performed, and variations in the level of monitoring pre- and peri-procedurally. The interscalene block remained the most popular choice of regional nerve block for arthroscopic shoulder surgery, with 10-14mL of ropivacaine being the local anaesthetic of choice (at concentrations of 0.75% or 0.375% predominantly). Ultrasound was used routinely by most block-respondents to assist block performance. Our findings showed a large proportion of block-respondents considered the procedure painful enough both to justify block performance and the routine provision of a discharge script for opiate analgesia. This survey has also highlighted numerous points that should be considered in any future review of college or other practice standards. These include the recommendations for optimal depth of consciousness for shoulder blocks, and the awareness of intralipid use and location in the event of an emergency. Importantly, the widespread deviation from current recommendations for standard monitoring during major regional anaesthesia should be investigated in future studies to ensure patient and anaesthetist safety is not being compromised.

## Supplementary Information

Supplementary Material 1.

## Data Availability

Data is provided within the manuscript or supplementary information files.

## Abbreviations

ANZCA: Australian and New Zealand College of Anaesthetists
NYSORA: New York School of Regional Anesthesia
SALHN HREC OFR: South Australia Local Health Network Human Research Ethics Committee Office for Research
PCA: Patient–Controlled Analgesia
ASA: Australian Society of Anaesthetists
QR: Quick Response (code)
LA: Local Anaesthetic
USA: United States of America
PS26: Position Statement 26 (ANZCA document)
ASRA: American Society of Regional Anesthesia and Pain Medicine
LAST: Local Anaesthetic Systemic Toxicity
WHO: World Health Organisation
