# Supplementary material for: A survey of anaesthetic management of regional anaesthesia for patients undergoing arthroscopic shoulder surgery in Australia
Source: BMC Anesthesiol. 2025 Nov 17;25:568. doi: 10.1186/s12871-025-03459-3 (PMC12625579; doi:10.1186/s12871-025-03459-3)
Supplement: Supplementary file 1 — Supplementary Material 1. [file 12871_2025_3459_MOESM1_ESM.pdf]

A survey of anaesthetic management of regional anaesthesia for patients undergoing arthroscopic shoulder surgery in Australia

Thank you for participating in this 5-minute survey. We will consider your voluntary participation as a mark of consent to partake in this clinical audit.
Your participation is anonymous and all results will be de-identified.

In order to partake in this survey, we require that both of the following criteria be met:

1. *You are a consultant of fellow anaesthetist*
2. *In the last year you have been the primary anaesthetist for ≥ 1 patient undergoing arthroscopic shoulder surgery*

If you do not meet these criteria, we thank you for your time but kindly request you do not participate.

The following questions will all refer to you and your personal choices regarding pain management in patients undergoing arthroscopic shoulder surgery. We ask for you to answer these questions with reference to your typical clinical practice. We understand there are some specific clinical situations that may lead you to deviate from normal practice, but we are not looking to address these with this survey.

The aim of this survey is to determine how a representative cohort of anaesthetists across Australia utilise regional anaesthesia for patients presenting for arthroscopicshoulder surgery.

<u>Part 1: Demographics</u>

Q1:
I am a:
- Consultant Anaesthetist
- Fellow

Q2:
My primary type of practice is:
"Primary" refers to the place in which you practice the majority of your working hours
- Public
- Private

Q3:
I have worked in clinical anaesthesia for:
For the purpose of this question, please include only years post-fellowship.
- 0-5 years
- 6-10 years
- 11-15 years
- 16-20 years
- 21-25 years
- >25 years

Part 2: Pre-procedural considerations

Q4:
In a patient presenting for 'shoulder arthroscopy +/- tendon repair' and no contraindications to regional anaesthesia, I would routinely place a nerve block:

- o True
- o False

Q5:
Please elaborate on your reasons for not routinely placing a nerve block for this patient group: (Free text)

Q6:
I routinely obtain written, informed consent from the patient for their nerve block:

- o True
- o False

Q7:
Regarding your preparation to perform a nerve block:
For each row, please choose one box

	Never	Sometimes	Often	Always
I ensure the block site/side is marked				
I personally mark the block site				
I perform a consent/side/site check				

Q8:
In routine circumstances, the level of sedation I use to perform my nerve block is:

- o No sedation
- o Light (talking, following command)
- o Moderate (easy to rouse, responsive to pain)
- o Heavy (difficult to rouse, may not respond to pain)
- o General anaesthesia

Q9:
In routine circumstances, while performing my nerve block:
For each row, please choose one box

	Never	Sometimes	Often	Always
I have a dedicated trained assistant with me				
I place an intravenous cannula prior to commencing the block				
I monitor oxygen saturations				
I monitor blood pressure				
I monitor electrocardiography				
I monitor respiratory rate or end-tidal carbon dioxide				

Q10:
I could easily and quickly access an appropriate dose of Intralipid 20% in all the institution(s) in which I work

- ○ Yes
- ○ No

Part 3: Peri-procedural considerations

Q11:
My block of choice for a patient undergoing arthroscopic shoulder surgery is:
(Please only select more than one option if you routinely perform > 1 block per standard patient)

- Interscalene block
- Superior trunk block
- Supraclavicular block
- Axillary nerve block
- Suprascapular nerve block
- Intercostobrachial nerve block
- Other

Q12:
My primary local anaesthetic agent for this block (or block combination) is:
This question will be repeated for those who use a mixture of local anaesthetics
(Please omit any local anaesthetic used for skin)

- Lignocaine
- Lignocaine with adrenaline
- Ropivacaine
- Bupivacaine
- Bupivacaine with adrenaline
- Levobupivacaine
- Other

Q13:
The concentration of my primary agent is:

- 2%
- 1%
- 0.75%
- 0.5%
- 0.375%
- 0.2%
- 0.125%
- Other concentration

Q14:
The volume (in mL) of my primary agent is: (free text)

Q15:
I routinely use a mixture of local anaesthetics for this block (or block combination)

- True
- False

Q16:
The additional local anaesthetic agent I use in mixture for this block (or block combination) is:

- Lignocaine
- Lignocaine with adrenaline
- Ropivacaine
- Bupivacaine
- Bupivacaine with adrenaline
- Levobupivacaine
- Other

Q17:
The concentration of my additional agent is:

- 2%
- 1%
- 0.75%
- 0.5%
- 0.375%
- 0.2%
- 0.125%
- Other concentration

Q18:
The volume (in mL) of my additional agent is: (free text)

Q19:
I routinely use adjuvants in my block injectate

- True
- False

Q20:
The adjuvants I use are:
(Select all that are applicable)

- Dexamethasone
- Fentanyl
- Morphine
- Buprenorphine
- Clonidine
- Dexmedetomidine
- Other adjuvant not listed

Q21:
What dose and concentration of adjuvant do you use? (free text)

Q22:
I use the following aids to assist in block performance:
For each row, please choose one box

	Never	Sometimes	Often	Always
Ultrasound				
Nerve stimulator				
Injection pressure monitor				

Q23:
For patient undergoing shoulder arthroscopic surgery, I leave a catheter in:
- o Never
- o Sometimes
- o Often
- o Always

Q24:
The person who performs the syringe injection is:
- o Me
- o My assistant
- o Other

Q25:
I use the following needle type:
- o 100mm echogenic
- o 50mm echogenic
- o Tuohy tip (epidural)
- o Hypodermic needle
- o Other

Part 4: Post-procedural considerations

Q26:
My above anaesthetic management is the same whether these patients are day surgery patients or overnight admission:

- True
- False

Q27:
For those patients who remain in hospital overnight I routinely prescribe a patient-controlled analgesia pump:

- True
- False

Q28:
I routinely provide a script for discharge opiates for patients having undergone post-shoulder arthroscopic surgery

- True
- False

Q29:
I routinely follow up all these patients either in person or over the phone:

- True
- False

Q30:
I have picked up a significant complication through patient follow up

- True
- False

---- End of survey -----
